# *In Silico* and *In Vitro* Screening of Natural Compounds as Broad-Spectrum *β*-Lactamase Inhibitors against *Acinetobacter baumannii* New Delhi Metallo-*β*-lactamase-1 (NDM-1)

**DOI:** 10.1155/2022/4230788

**Published:** 2022-03-10

**Authors:** Aparna Vasudevan, Dinesh Kumar Kesavan, Liang Wu, Zhaoliang Su, Shengjun Wang, Mohan Kumar Ramasamy, Waheeta Hopper, Huaxi Xu

**Affiliations:** ^1^International Genomics Research Center (IGRC), Jiangsu University, Zhenjiang 212013, China; ^2^Department of Immunology, School of Medicine, Jiangsu University, Zhenjiang 212013, China; ^3^Interdisciplinary Institute of Indian System of Medicine, SRM Institute of Science and Technology, Tamil Nadu, India; ^4^Department of Biotechnology, School of Bioengineering, Faculty of Engineering & Technology, Kattankulathur Campus, SRM Institute of Science & Technology, India

## Abstract

Antibiotic resistance is one of the significant problems globally; there is an increase in resistance with introducing every new class of antibiotics. Further, this has become one of the reasons for arising of new resistance mechanisms in *Acinetobacter baumannii*. In this study, we have screened natural compounds as a possible inhibitor against the NDM-1 *β*-lactamase enzyme from *A. baumannii* using a combination of *in silico* methods and *in vitro* evaluation. The database of natural compounds was screened against NDM-1 protein, using Glide docking, followed by QM-polarised ligand docking (QPLD). When the screened hits were validated *in vitro*, withaferin A and mangiferin had good IC_50_ values in reducing the activity of NDM-1 enzymes, and their fractional inhibitory concentration index (FICI) was ascertained in combination with imipenem. The withaferin A and mangiferin-NDM-1 docking complexes were analyzed for structural stability by molecular dynamic simulation analysis using GROMACS for 100 ns. The molecular properties of the natural compounds were then calculated using density functional theory (DFT). Withaferin A and mangiferin showed promising inhibitory activity and can be a natural compound candidate inhibitor synergistically used along with carbapenems against NDM-1 producing *A. baumannii*.

## 1. Introduction

The worldwide increase in carbapenem resistance among Gram-negative bacteria has become a significant clinical concern. *Acinetobacter* is one such species that showed high resistance after just four years of its identification in 1971 and as early as the 1990s. They were resistant to imipenem, the trusted drug of choice [1] [2]. Resistance to carbapenems in *A. baumannii* (carbapenem-resistant *A. baumannii* (CRAB)) can itself make them highly resistant. Apart from the intrinsic resistance determinants, oxacillinases (Ambler Class D) and Metallo *β*-lactamases (Ambler Class B) are major contributors to resistance against carbapenems [3].

There are three subclasses under the Metallo *β*-lactamases, B1, B2, and B3; the New Delhi Metallo-*β*-lactamase 1 (NDM-1) belongs to B1. NDM-1 is an emerging concern among the heterogeneous group of carbapenemases, first described from *Klebsiella pneumoniae*, *Escherichia coli*, *Pseudomonas aeruginosa*, *A. baumannii*, and, more recently, Enterobacteriaceae [4] [5] [6].

NDM-1-positive strains have been rapidly and widely spreading in many countries among *A. baumannii* strains [7]. NDM-1-positive strains are resistant to fluoroquinolones, aminoglycosides, and *β*-lactams (especially carbapenems) while being susceptible to colistin and sometimes to tigecycline. NDM-1 resistance dissemination occurs by transferring resistance plasmids with the *bla*_NDM-1_ gene, making them a severe clinical and public health concern [7].

**Figure 1:**
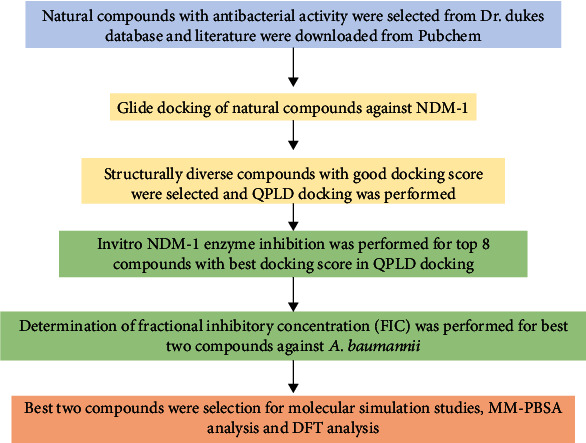
Flow chart of virtual screening, filtering, and experimental screening strategy for identifying NDM-1 inhibitors.

The crystal structure of NDM-1 reveals two zinc ions, which are essential for cleaving the C-N bond in the *β*-lactam ring of the antibiotic, thereby inactivating them. NDM-1 structure comprises lysine-rich residues that help protonate lactam nitrogen in *β*-lactam antibiotics [8]. MBLs are resistant to commercially available *β*-lactamase inhibitors, such as clavulanic acid, sulbactam, tazobactam, and avibactam, and there are no commercially available MBL inhibitors meant for clinical use [9]. Increased resistance to carbapenem in *A. baumannii* is often seen with a high mortality rate. The current management of infections and outbreaks due to MDR *A. baumannii* requires a combination of medical interventions, antibiotic stewardship, environmental, and prevention of resistance dissemination. There is an urgent need for newer ways to combat the infections; one is to synergistically enhance the bioactivity of the available drugs with inhibitors of resistance determinants [10]. The purpose of this research is to search for natural compounds that can inhibit acquired *β*-lactamases, which contribute to carbapenem resistance in *A. baumannii*, specifically NDM-1 Metallo-lactamase. The workflow for the study is given as in Figure 1. A preliminary computational method was used to identify a subset of compounds from a database of natural compounds by predicting their binding mode against the target proteins from *in silico* molecular docking experiments and density functional theory (DFT). The top hits were validated by molecular dynamic simulation studies and *in vitro* enzyme inhibition assay, and fractional inhibitory concentration and *in silico* ADME properties were predicted.

## 2. Materials and Methods

### 2.1. Protein and Ligand Preparation

The protein sequence information of NDM-1 from *A. baumannii* was retrieved from NCBI (accession number: ALD19783). The 3D structure of NDM-1 protein was modeled using Swiss Prot 3D modeling server [11]. Homology modeling commonly results in unfavourable bond lengths, bond angles, torsion angles, and contacts in protein models. As a result, it was critical to minimize energy to standardize local bond and angle geometry and relax near contacts in the geometric chain. The 3D model of NDM-1 was validated after optimization using PROCHECK [12], ERRAT [13], and VERIFY3D [14] programs from the Structural Analysis and Verification Server (SAVES). PROCHECK was used to assess the stereochemical consistency of the protein structure, and the VERIFY3D program was used to evaluate the 3D protein structure by analyzing the compatibility of an atomic model (3D) with its amino acid sequence (1D).

All analyses were performed using Schrödinger LLC-Maestro version 10.2. The modeled 3D protein and PDB structures were minimized with OPLS-2005 force field using protein preparation wizard in Maestro. Using OPLS-3e force field in Prime program, missing hydrogens atoms, missing side chain atoms, and missing loops were inserted. Restrained minimization was used to minimize hydrogen atoms and heavy atoms, and a receptor grid generation module was used to generate a grid using the binding site residues of modeled NDM-1. The natural compounds collected with known antibacterial bioactivity from plants were obtained from literature and Dr. Duke's Phytochemical and Ethnobotanical Database [15]. The structures of the compounds were retrieved from the PubChem database and subjected to ligand preparation. LigPrep module assigns proper bond orders and corrects the protonation and ionization states of the compounds; after that, each ligand's tautomeric and ionization states were formed. Default values were used for pre- and postprocess minimization; a maximum of 100 conformers were generated per structure. Each minimized conformer was filtered through an 11.4 kcal/mol (50 kJ/mol) relative energy window with a maximum atom deviation of 2.00 Å.

### 2.2. Glide XP Docking

XP Glide module was used to perform molecular docking studies with the natural compound database against NDM-1. The XP docking score was enhanced by several factors such as the addition of large desolvation penalties to both ligand and receptor; a specific structural motif that contributes to binding affinity; and sample and an algorithm for scoring functions. The XP docking scoring functions include Ecoul (Coulomb energy), EvdW (van de Waals energy), Ebind (components favouring binding), and Epenalty (components impeding binding) [16]. The top-ranking poses were chosen based on the Glide ratings for further analysis. Finally, the top eight compounds were selected for further investigation.

### 2.3. QPLD Docking

QM-polarised ligand docking (QPLD) method was used to dock all ligand molecules with modeled NDM-1 protein. QPLD docking method is used to improve docking scores by calculating quantum mechanics and molecular mechanics (QM/MM) approach using QSITE program coupled with Jaguar for QM region and the IMPACT molecular modeling code for MM region [17]. The QM/MM energy is calculated as the complex's Coulomb–van der Waals force, calculated from the ligand's electrostatic potential energy. This is derived from a single-point calculation for the QM region using density functional theory using the 6-31G∗/LACVP∗ basis set, B3LYP density functional, and “Ultrafine” SCF accuracy level (iacc = 1 and iacscf = 2) [18]. Briefly, three steps were included in the protocol: first, generating the best pose for ligand docking using standard precision (SP) scoring mode followed by XP refinement; second, initial partial charges on ligand atoms were removed, and QM-ESP charges were calculated from the electrostatic potential energy surface of the ligand, generated from a single-point calculation using B3LYP/6-31G∗ level within the protein environment and density function for the QM region; and third, the ligands were redocked in the most energetically favourable pose using Glide standard docking for QPLD refinement, and then, the level of quantum–mechanical treatment was set at Fast mode [19].

### 2.4. DFT Calculations

The density functional theory (DFT) analysis was performed for the natural compounds. The minimized ligands obtained from LigPrep were used for DFT calculations using the Jaguar panel of Maestro Schrödinger. Recognized conformers were studied by DFT using the Jaguar module in Maestro. DFT calculations were performed to determine the electronic molecular properties, such as electron density, molecular electrostatic map, and frontier molecular orbital density fields, revealing biological activity and molecular characteristics (lowest and highest occupied molecular orbital). These compounds were measured using DFT using a Jaguar module depending on the solvation condition. Using the 491 6-31G∗∗ basis set standard, the conformers were analyzed using the Lee-Yang–Parr correlation functional (B3LYP) and Becke's three-parameter exchange potential [20]. With single-point calculations, the implicit redemption model of the Poisson–Boltzmann Finite (PBF) was calculated. Dipole moment, highest occupied molecular orbital (HOMO), lowest unoccupied molecular orbital (LUMO) energy, and lowest unoccupied molecular orbital (MESP) were used to calculate the molecular electrostatic properties. The electrostatic potentials were calculated using the molecule's van der Waals (vdW) contact surface region. The colour-coded surface values showed the positive electrostatic potentials and total molecular size. The most positive electrostatic potential regions are indicated by the darkest blue colour, whereas the most negative electrostatic potential regions are indicated by the deepest red colour [19, 21].

### 2.5. ADME Analysis

Absorption, distribution, metabolism, and excretion (ADME) properties of natural compounds were calculated using the QikProp module in Maestro. QikProp module predicts the several pharmaceutically relevant properties and other physically significant ADME property descriptors for the ligand. The essential properties for drug-likeness, like log P, human oral absorption, and Lipinski's rule of 5, were calculated in the QikProp module [22].

### 2.6. Molecular Simulation

The docked protein-ligand complex of apoproteins and ligand complexed NDM-1 were simulated to study the stability using molecular dynamic simulation analysis software GROMACS 5.16 [23]. Apo proteins and protein-ligand complexes were simulated for a 100 ns time scale to get an insight into the conformational changes of atoms in protein-ligand complexes in the dynamic environment. Using the PRODRG server and GROMACS tools, the topologies were generated for all the ligands in the complexes. The structure of protein complexes was relaxed and hydrated using simple point charge (SPC) water with periodic boundary conditions. Genion tool in GROMACS was used to add Na+ and Cl− to the system to neutralize the system. The Verlet cut-off scheme was used to perform an energy minimization iteration. The Ewald method was used to calculate the long-range electrostatic and van der Waals interactions with a cut-off of 1.0 and 1.2 nm. The simulation was performed after equilibrating the system to the room temperature, pressure, and constant number of particles (NPT), and a simulation of 100 ns was performed [24]. The trajectories were used for various dynamic analyses, such as root mean square deviation (RMSD), root mean square fluctuation (RMSF), and the radius of gyration (Rg); a number of hydrogen bonds and SASA by different inbuilt scripts of GROMACS after the 100 ns MD simulation were completed. MM-PBSA binding free energy of receptor-ligand complexes was calculated using the molecular mechanics/Poisson–Boltzmann surface area (MM/PBSA) process, which uses MD simulation trajectories and is one of the most widely used methods in this field [25].

### 2.7. Molecular Docking of Natural Compounds with Other Types of *β*-Lactamases

The natural compounds were taken for Glide docking analysis against other different types of *β*-lactamases from various organisms. The study was done to analyze the binding efficiency of these compounds against non-NDM-1 *β*-lactamases. The *β*-lactamase, ADC-7 (PDB ID: 6PWL, *A. baumannii*), AmpC (PDB ID: 1KDW, *E. coli*), CTX-M-15 (PDB ID: 4HBT, *E. coli*), KPC-2 (PDB ID: 3RXX, *K. pneumoniae*), SHV-1 (PDB ID: 3MXR, *K. pneumoniae*), TEM-1 (PDB ID: 1TEM, *E. coli*), OXA-24/40 (PDB ID: 6MPQ*, A. baumannii*), and VIM-2 (PDB ID: 2YZ3, *Pseudomonas putida*) were selected for the Glide docking studies. The previously used protein preparation and Glide protocol in Sections 2.1 and 2.7 were followed for *β*-lactamase protein and the four natural compounds.

### 2.8. *In Vitro* NDM-1 Enzyme Inhibition Assay

The NDM-1 enzyme inhibition assay was carried out spectrophotometrically using nitrocefin as a chromogenic substrate as described previously [26] [27]. Briefly, the known inhibitors (EDTA (Aladdin, China, E299201) and substrate (nitrocefin (Aladdin, China, N163020)) and natural compounds were dissolved in DMSO. Recombinant NDM-1 enzyme (RayBiotech Life ID: 230-00554-500, Georgia) at a concentration of 1 nM was supplemented with 10 *μ*M ZnSO4, incubated with various concentrations (10-100 *μ*M) of shortlisted compounds from computational screening (10 *μ*L of DMSO as negative control) for 10 min at 30°C temperature. Nitrocefin was then added at a final concentration of 60 *μ*m, and the absorbance was measured at 490 nm at 30°C temperature using a multimode reader (BioTek Synergy, United States). The IC_50_ values were determined thrice by fitting the concentration dependence of residual enzyme activity to the nonlinear regression using GraphPad Prism 5.03.

#### 2.8.1. Determination of Fractional Inhibitory Concentration (FIC)

The combined effect of the natural compounds and imipenem (Zhejiang Hisun Pharmaceutical, China) was done using the checkerboard synergy titration assay in a 96-well microtitre plate [28]. Imipenem was tested at concentrations of eight dilutions lower than their MIC and two dilutions higher than the MIC. MIC concentration of imipenem (4x) was added in all wells under column 1 of the 96-well checkerboard panel for each concentration of natural compounds (one each row). The compounds were serially diluted at different subinhibitory concentrations, and each concentration was added to the corresponding rows. The bacterial strain (PAN resistance *A. baumannii* clinical isolate (strain number: P-29) was adjusted to 0.5 McFarland's standard and added to each well. The plates were incubated at 35°C and read at 600 nm using a BioTek microplate reader. The compound's fractional inhibitory concentration (FIC) index was calculated as given by the formula (MIC of A in combination with B/MIC of A alone) + (MIC of B in combination with A/MIC of B alone).

A FIC = 0.5 for a compound was taken as synergistic, FIC > 0.5 − 4 was additive/indifferent, and FIC > 4 was taken as antagonistic.

## 3. Results and Discussion

### 3.1. Homology Modeling of NDM-1

The Ramachandran plot was used to evaluate the reliability of the NDM-1 3D model using PROCHECK software (Suppl. Figure 1). The predicted 3D model's Ramachandran plot showed that 89.7% of residues were in the most favourable region, while 9.8% were in the allowed region, suggesting that the predicted model is of good quality. The “overall quality factor” for nonbonded atomic interactions is given by ERRAT scoring; the higher the scores, the better the quality. The commonly accepted range for a high-quality model is >50. The overall quality factor predicted by the ERRAT server for the current 3D model was 84.40, and the VERIFY3D server predicted that 96.68% of the residues in NDM-1 had an average 3D-1D score > 0.2, indicating that the model was valid.

**Figure 2:**
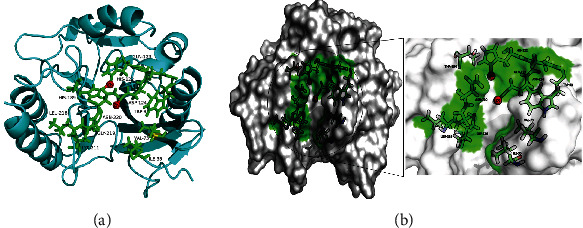
The active site region of NDM-1 protein. Panel (a) gives the cartoon model representation of NMD-1, and panel (b) shows the active site amino acid residues in the cavity of NDM-1 receptor. The active site amino acids are shown as green carbon atom stick and surface representation and Zn ions shown as red color sphere, whereas the protein is depicted in grey color surface.

The active site of NDM-1 includes residues ILE35, MET67, VAL73, TRP93, HIS120, GLN123, HIS122, ASP124, ASN220, GLY219, LYS211, LEU218, HIS189, and HIS250, as shown in (Figure 2) [5]. Crucial for bioactivity of Class B *β*-lactamases are zinc ions; there are two zinc ions surrounded by HIS120, HIS 122, ASP124, HIS189, CYS208, and HIS250 in the NDM-1 protein. The *β*-lactam ring interacts with two zinc ions, LYS211, a conserved residue ASN 220, ASP123, and ASP124, contributing to binding and orientation [6].

### 3.2. Molecular Docking Analysis

The literature and Dr. Dukes database selected the natural compounds with only antibacterial activity, and a database was created for this study. A total of 168 phytochemicals were shortlisted using Glide docking against NDM-1 protein. The natural compounds with the maximum docking score and binding mode in the active sites of the proteins were prioritized, and the top eight compounds interacting with active site residues of NDM-1 were chosen for further analysis (Table 1).

**Figure 3:**
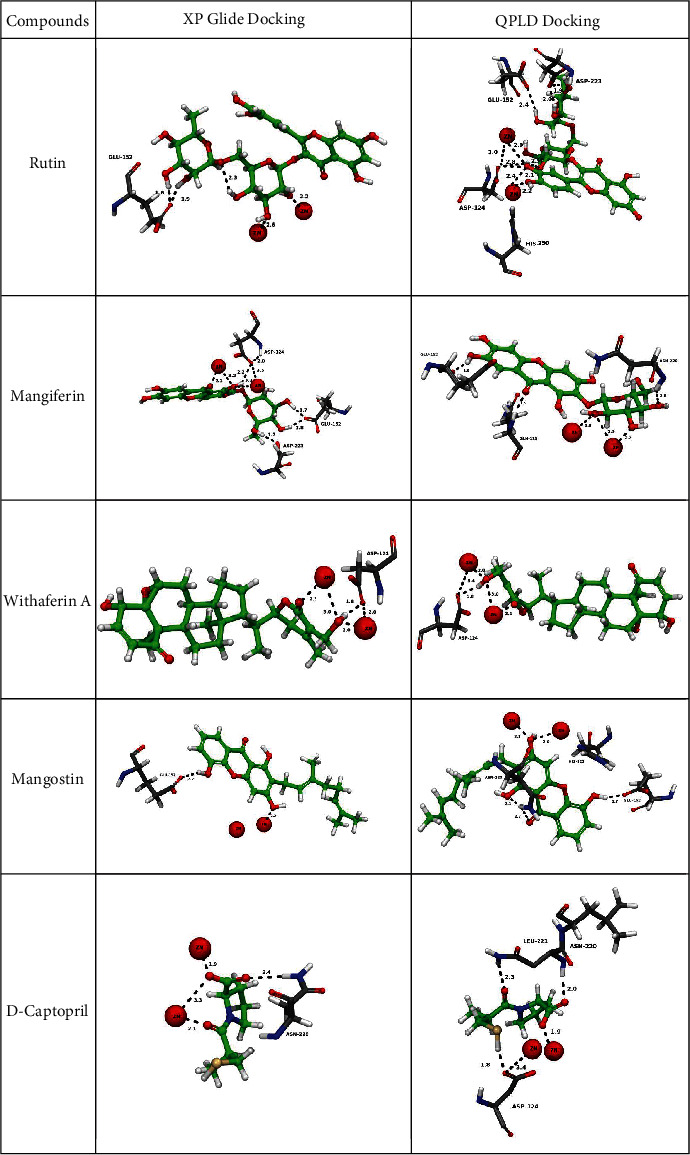
Docking interactions of natural compounds with active site residues of NDM-1 protein. Residues lining in the active site are shown as grey colour capped sticks. Ligands are green-coloured. Zn atoms are represented in red colour spheres. Hydrogen bonds are represented by black dashed lines.

The results of molecular docking and molecular interactions provide additional insights into selective interactions of natural compounds with NDM-1, as shown in Figure 3 and Suppl. Figure 2. The natural compound database was docked into the active site of NDM-1 based on the docking score priorities; compounds were selected for further studies. Based on the hydrogen-bonding interactions and docking score, the top 8 compounds with structural diversities and binding mode were chosen. Figure 3 shows the protein-ligand complexes and their hydrogen bond interactions.

The enzyme inhibition assay with *β*-lactamase and NDM-1 enzyme was performed with 8 compounds, mentioned in Table 1. Of the eight, only two compounds (withaferin and mangiferin) showed the highest activity, and two compounds showed moderate activity (rutin and mangostin) (explained in Section 3.5). The four compounds were further analyzed for docking interaction with NDM-1. Four compounds, rutin, mangiferin, withaferin A, and mangostin, were found to have good Glide score for NDM-1 (-9.44, -9.12, -5.12, and -6.54 kcal/mol, respectively) and the Glide score for the known inhibitor D-captopril (-5.52), respectively. Molecular docking analysis showed that all four selected compounds interacted with NDM-1 catalytic amino acid residues through hydrogen bonding and hydrophobic interactions.

**Figure 4:**
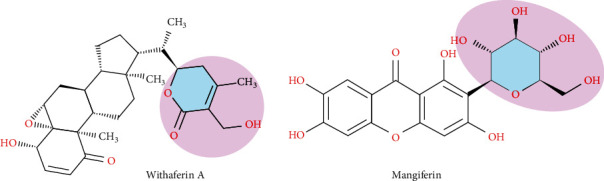
Structure representing the bioactive moiety of withaferin A and mangiferin against *β*-lactamase.

Withaferin A, a terpenoid containing four cycloalkane ring structures, three cyclohexane rings, and one cyclopentane ring, is isolated from *Acnistus arborescens* and *Withania somnifera* [29]. Recent research has shown that withaferin A has anticancer, adaptogenic, antistress, anticonvulsant, immunomodulatory, and neurological effects. It is a natural proteasome inhibitor, an apoptosis inducer by inhibiting the topoisomerase-I DNA complex, and a mitotic poison with antiangiogenic properties [30]. Withaferin A showed a Glide score of -5.12 and -6.21 kcal/mol and Glide energy of -36.50.15 and -43.02 kcal/mol from QPLD, respectively, and binding affinity with NDM-1. In both XP and QPLD docking analyses, withaferin interacted strongly with ASP124 (1.8 Å) and Zn+ and formed hydrophobic interactions with ILE35, VAL73, MET67, and TRP93 (Figure 3 and Suppl. Figure 2). A pharmacophore of withaferin A involves the 4-hydroxy-5,6-epoxy-22-en-1-one moiety, and its unsaturated lactone has been identified as essential for cytotoxic activity by structure-activity relationship studies [31]. In this study, we found that (6S)-6-ethyl-3-(hydroxymethyl)-4-methyl-5,6-dihydropyran-2-one region of withaferin A formed hydrogen bond with active site amino acids in NDM-1. This showed that (6S)-6-ethyl-3-(hydroxymethyl)-4-methyl-5,6-dihydropyran-2-one region might be important in the biological activity of the withaferin A against *β*-lactamase (Figure 4).

Mangiferin is a xanthone found in higher plants and various parts of the mango crop, including the peel, stalks, leaves, barks, kernel, and stone. It is a promising antioxidant with a long list of health benefits, including antiviral, anticancer, antidiabetic, antioxidative, antiaging, immunomodulatory, hepatoprotective, and analgesic properties [32].

Mangiferin docked with NDM-1 protein gave a Glide score of -9.12 and -10.62 kcal/mol, and Glide energy of -57.25 and -52.76 kcal/mol was observed by XP and QPLD analysis. Mangiferin formed hydrogen bond between 9H-xanthen-9-one and D-glucitol region and active site amino acids ASP223 (2.0 Å), GLU152 (2.4 Å), ASP124 (2.5 Å), and Zn+ in XP docking and GLU152 (1.9 Å), GLN123 (1.9 Å), and ASN220 (2.0 Å) in QPLD docking (Figure 3 and Suppl. Figure 2). The 1,5-anhydroglucitol region of mangiferin formed hydrogen bonds with active site amino acids of NDM-1, indicating the possible biological activity of mangiferin (Figure 4).

Mangostin is a significant xanthone found in the *Garcinia mangostana* Linn (mangosteen tree's) fruit pericarps, bark, and dried sap. Mangostin has a wide range of pharmacological effects, including antioxidant, anticancer, and anti-inflammatory effects [33]. From docking analysis with NDM-1, Glide scores of -6.54 and -7.76 kcal/mol and Glide energy of -40.17 and -44.45 kcal/mol were observed in the XP and QPLD. The hydrogen bonds were formed between 9H-xanthene region of mangostin and active site amino acids GLU152 (2.2 Å) in XP docking and ASN220 (2.1 Å) and GLU152 (1.7 Å) in QPLD docking (Figure 3). In QPLD docking, 9H-xanthene region of mangostin formed PI–PI cation interaction with HIE122 and a strong interaction between Zn+ in the active site (Figure 3 and Suppl. Figure 2).

A flavonol glycoside, rutin (quercetin-3-rutinoside), is between quercetin and *α*-l-rhamnopyranosyl-(16)—d-glucopyranose [34]. Rutin contains flavonolic aglycone quercetin and disaccharide rutinose [35], the hydroxy (-OH) groups in the sugar moiety aid in the formation of H-bond interactions between amino acid residues. Docking results of NDM-1 and rutin are shown in Table 1 and (Figure 3 and Suppl. Figure 2). Rutin with NDM-1 showed a Glide score of -9.44 and -9.31 kcal/mol and Glide energy of -59.59 and -88.67 kcal/mol in XP docking and QPLD docking methods. In XP docking, rutin interacted with GLU152 (1.6 Å), ASP124 (2.4 Å), GLU152 (2.4 Å), ASP223 (2.0 Å), and Zn+ ions in QPLD. In both docking methods, rutin strongly binds to the two Zn+ ions. And it also formed PI–PI interaction between its quercetin region and HIS250 residue in the active site.

The Structural Interaction Fingerprints (SIFt) algorithm helps find the types of interactions in a binding site by locating amino acids around bound ligands and residue types. SIFt was analyzed using residue features such as backbone, side chain, hydrophobic, aromatic, acceptor, donor, polar, and charged groups in the neighbourhood of the ligand's binding site. Withaferin A interacts with most of the side chains in the active site except GLY219 (Table 2). Two hydrophobic interactions were found in the ILE35 and TRP93. It also forms polar interactions with HIS122, ASP124, HIS189, SER217, ASN220, and HIS250. Withaferin A forms aromatic (TRP93), acceptor (ASP124), and charged (ASP124) interaction with NDM-1 binding site. GLY219 and HIS250 form backbone interaction with withaferin A. In SIFt analysis, mangiferin shows side chain and polar interaction with most of the amino acids in the binding site in the NDM-1 (Table 3). It forms hydrophobic interaction with ILE35 and forms acceptor and charged interaction with ASP124, GLU152, and ASP223. The backbone amino acid interaction between mangiferin is found in the HIS122, GLN123, and GLY219, respectively.

### 3.3. Validation of XP Docking Protocol

The validity of the docking protocol was performed by redocking the X-ray crystal structure ligand (i.e., D-captopril) at the active site of NDM-1. RMSDs and amino acid interactions between the docked pose and the crystal structure pose of D-captopril are as shown in the suppl. Figure 3. We found that D-captopril redocked into the active site of NDM-1, which is similar to the crystal structure.

### 3.4. Binding Free Energy Calculation

The MM-GBSA was used to determine the effect of solvent and free energy on the interaction between natural compounds and NDM-1 proteins. From Glide XP and QPLD docking, ligand-bound complexes were obtained, and MM-GBSA calculation was performed using surface area energy, solvation energy, and energy minimization of the protein-ligand complexes. The MM-GBSA of 4 natural compounds and known inhibitors are shown in Table 4. And for NDM-1 docked poses, Prime MM/GBSA (DGBind) ranges from -32.20 and -33.49 kcal mol (withaferin A), -23.92 and -21.05 kcal mol (mangiferin), -15.74 and -13.92 kcal mol (mangostin), -24.32 and -27.18 kcal mol (rutin), and -11.93 and -18.57 kcal mol (sulbactam) in XP and QPLD docking. The results showed that nonsolvation terms (Glipo), van der Waals (GvdW), and covalent energy (Gcovalent) are more promising contributors for ligand binding. The binding free energies of these known inhibitors do not change significantly.

### 3.5. Determination of the Inhibitory Activity of Candidate Natural Compounds against NDM-1

**Figure 5:**
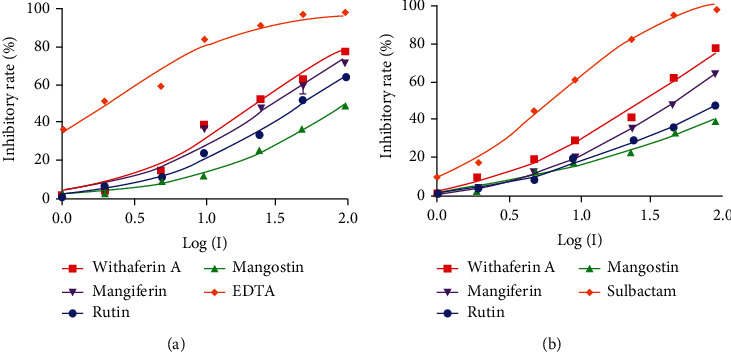
Effect of natural compounds and known inhibitors on (a) NDM-1 and (b) *β*-lactamase inhibition activity.

The IC_50_ values were calculated for natural compounds and known inhibitors under controlled experimental conditions. The percent inhibitory curves for the candidate natural compounds with efficacy for NDM-1 and *β*-lactamase enzyme are given in Figures 5(a) and 5(b). The natural compounds were incubated with NDM-1 enzyme, and the substrate, nitrocefin, and hydrolysis of the substrate were monitored at 490 nm OD.

#### 3.5.1. NDM-1 Enzyme Inhibitory Activity Assay

Of the eight natural compounds (at concentrations 10-100 *μ*M) tested for the NDM-1 enzyme inhibitory activity assay, only four compounds showed 50% maximum inhibitory activity suggesting they could be potential inhibitors. The addition of withaferin A, mangiferin, rutin, and mangostin to the enzyme assay reduced the NDM-1 enzyme activity (reduction in the hydrolysis of nitrocefin) significantly with IC_50_ of 24.03 ± 2.92 *μ*M for withaferin A, mangiferin 30.6 ± 4.41 *μ*M, and rutin 48.94 ± 3.74 *μ*M and minimal reduction with mangostin 97.48 ± 5.59 *μ*M. The IC_50_ values of other compounds were more than 100 *μ*M, indicating that these compounds can have minimal or no inhibitory effect on NDM-1 and the IC_50_ of the known NDM-1 enzyme inhibitor EDTA was 2.081 ± 0.296 *μ*M.

#### 3.5.2. *β*-Lactamase Enzyme Inhibitory Activity Assay

The eight candidate natural compounds identified by *in silico* analysis against NDM-1 were also tested for *β*-lactamase inhibition assay. Of the eight, the addition of compounds withaferin A, mangiferin, rutin, and mangostin in the assay decreased *β*-lactamase activity significantly with IC_50_ of 32.83 ± 2.59 *μ*M for withaferin A and mangiferin 58.02 ± 2.08 *μ*M. A very minimal inhibition of the enzyme activity was observed in the presence of rutin 127.4 ± 15.9 *μ*M and mangostin 136.1 ± 37.1 *μ*M, while that of the known *β*-lactamase inhibitor sulbactam was 7.244 ± 0.392 *μ*M.

#### 3.5.3. Augmentation of Antimicrobial Effect of Imipenem by the Withaferin A and Mangiferin

Every new development in drug discovery is the challenge of resistance that competes equally. Making a comeback for the already available drugs differently is a more approachable method to combat the resistance development. Combination therapy is not a new avenue; combination drugs have been routinely used to treat life-threatening illnesses. Plant natural compounds are weak antimicrobials; however, as seen in *in vitro* studies, the synergizing effect has proven effective when used together.

The compounds, withaferin A and mangiferin, showed good enzyme inhibition activity against NDM-1 and were checked for their mechanism of action (potential augmentation effect) with imipenem against the carbapenem-resistant *A. baumannii* strain. The subinhibitory concentrations of the natural compounds were used to test the synergy along with imipenem, and the results were given as fractional inhibitory concentration values.

The checkerboard method was used to design the dilution panel, with twofold concentrations above the MIC of the antibiotics and MIC concentration and fivefold below the MIC. The 96-well microtitre plate was used for the above study, and the effects of the compounds on the MIC of imipenem are classified as synergistic, indifferent, or additive based on the FIC index as given in Table 5. Four natural compounds shortlisted from the enzyme inhibition assay were tested for synergy effect with imipenem, of which withaferin A and mangiferin had a potentiating effect on imipenem.

Withaferin A (128 mg/L) exhibited highly significant synergistic/potentiating effect with imipenem tested against carbapenem-resistant *A. baumannii* clinical strain (FIC index of 0.3125) followed by mangiferin, (128 mg/L) with an FIC index of 0.625. It is not surprising to see the synergistic potential of withaferin A, which has proven potentiating effect with anticancer drugs [36]. *Withania somnifera*, the source plant, has numerous therapeutic benefits. A previous structure-activity study by Moujir et al. has explored the antimicrobial properties and the functional groups involved in their potency [37]. Kannan and Kulandaivelu reported the antibacterial activity of Withaferin A activity towards both Gram-negative and Gram-positive bacteria, including *Bacillus subtilis*, *E. coli*, and *Staphylococcus aureus* [38]. *Anemarrhena asphodeloides*, a plant used in traditional Chinese medicine and whose main active component is mangiferin, has been found to have antiviral and antibacterial activities [39]. Mangiferin has many properties and is traditionally used in many countries; in Cuba, they are available with the brand name Vimang® and in Sri Lanka as Salaretin® [40]. Mangiferin has also been previously reported to be a good potentiator, along with many antibiotics proving to be effective for therapy [41]; this coincides with the findings from our current view of mangiferin in aiding the efficacy of available antibiotics. Mangiferin has been shown to have antibacterial activity against two *S. aureus* and *Salmonella typhi* [42] [41]. Mangiferin and its derivatives showed antibacterial and antifungal action against *Bacillus pumilus*, *Bacillus cereus*, *Salmonella virchow*, and two fungal species, *Thermoascus aurantiacus* and *Aspergillus flavus* [43].

### 3.6. ADME

*In silico* predicted ADME (absorption, distribution, metabolism, and excretion) properties of four natural compounds were done using the QikProp (Table 6). Drug kinetics and tissue exposure, which are essentially determined by their ADME properties, affect a drug's pharmacological activity and efficacy. Natural compounds were predicted and analyzed for approximately 13 physically important descriptors and pharmacologically active properties. The analysis of predicted ADME properties shows QPlog QPlogPo/w QPpolrz, QPlogS, QPlogPw, QPlogPoct, QPlogKp, QPlogPC16, and Khsa in the allowed range. The physiochemical properties of the natural compounds were within the allowed ADME range. Molecular weights of these natural compounds range between 380 and 610 g/mol, and each comprises 1–6 H-bond donor groups, 2-10 H-bond acceptor groups, and 5-15 rotatable bonds. The identified compounds' total polar surface area (PSA) was within the 92-273 Å^2^ range. And drug-like properties like QPpolrz, aqueous solubility (QPlogS), hexadecane/gas (QPlogPC16), octanol/gas (QPlogPoct), water/gas (QPlogPw), octanol/water (QPlogPo/w), skin permeability (QPlogKp), and Khsa serum protein binding (QPlogKhs) were shown to be in the allowed region. All the values for natural compounds come under the desirable range making it a suitable drug candidate.

### 3.7. Molecular Dynamic Simulation Results

#### 3.7.1. RMSD

**Figure 6:**
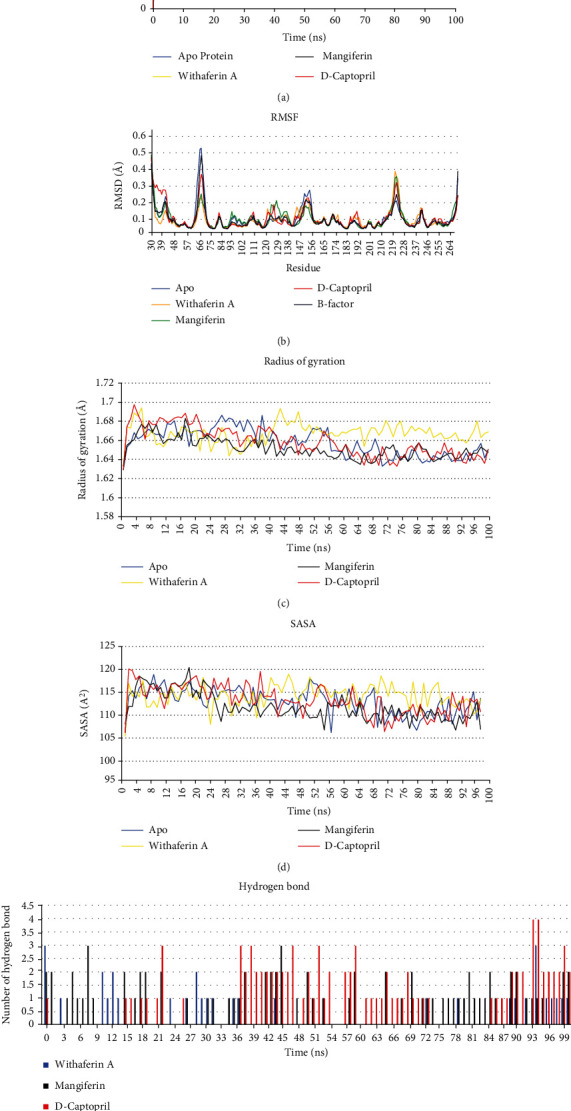
RMSD, RMSF, RG, SASA, and H-BOND of the backbone atom of the NDM-1 protein simulated over a time period of 100 ns.

After the QPLD docking study, molecular simulations of all the protein-ligand complexes and apoproteins were carried out for a period of 100 ns. The stability and conformational distribution of the ligands in the receptor protein were calculated using generated RMSD trajectories. For NDM-1, at the initial simulation phase, the backbone of all complexes was increased between 0 and 3 ns. In the apo NDM-1 protein, an increased fluctuation was observed at 43 ns, RMSD of 0.27 nm; after this, a stable deviation was observed throughout the simulation time, with an overall RMSD of 0.25 nm (Figure 6(a)). NDM-1 bound withaferin and D-captopril had a maximum deviation at around 40 ns with RMSD of 0.2 nm and 0.25 nm, respectively, whereas mangiferin RMSD reached a maximum of 0.3 nm before 20 ns simulation. RMSD trajectories of NDM-1, when analyzed together with known inhibitors and natural compounds, showed that after an initial fluctuation, a successful steady confirmation with minimal deviation was maintained throughout the simulation.

#### 3.7.2. RMSF

The conformation and stability of Apo and protein-ligand complexes were determined by their amino acid residues. It was perceived from the RMSF plot that NDM-1 and ligand complex attained the overall low RMSF than apoprotein. The plot suggested the stable binding of all three ligands and residues interacting with all three ligands having low flexibility compared with apoprotein fluctuation data. The resultant RMSF profile of NDM-1 apo and ligand complex showed fluctuations in the range of 0.1 to 0.25 and 0.1 to 0.52 nm at the protein's catalytic site and noncatalytic sites. We have calculated the B-factor for Apo protein and compared it with RMSF of a protein-ligand complex.

In NDM-1, high fluctuation (0.3 to 0.52 nm) was observed in the loop region of GLU30, ILE31, MET39, ASP66, MET67, PRO68, GLY69, PHE70, and ARG270 in apo and ligand-bounded complexes of NDM-1. These regions belong to the loop region in the NDM-1 and had fluctuations in both apo and ligand-bound complexes. In D-captopril–NDM-1 complex, high fluctuation was observed in the N-terminus loop region of GLU30, THR34, GLN37, and THR41 with RMSF around 0.25 to 0.46 nm. In ligand-bounded NDM-1 complex, slight fluctuations were observed in the loop region of ASN220, LEU221, GLY222, ASP223, ALA224, and ARG270 RMSF of 0.2 to 0.38 nm (Figure 6(b)). In this study, NDM-1 ligand-bounded complexes had no significant fluctuations at the ligand-binding site in the protein-ligand complexes compared with the Apo protein (Figure 6(b)). B-factor RMSF from the crystal structure and the simulated RMSF of NDM-1 were highly correlated throughout 100 ns.

#### 3.7.3. The Radius of Gyration (Rg)

The radius of gyration (Rg) indicates the size and compactness of the protein. The Rg values of NDM-1 complexes were 1.7 nm at the initial state. The fluctuation of Rg of apo and ligand-bounded NDM-1 was observed initially, and it was stabilized after five ns. For apoprotein, Rg fluctuation was observed in 27 and 38 ns, and for withaferin A–NDM-1 complex, a slight increase in fluctuations was there at 43 and 48 ns, and the system was stable after that, still 100 ns. For D-captopril–NDM-1 complex, there was high fluctuation initially, and it was stabilized after 20 ns till 100 ns. For mangiferin–NDM-1 complex, overall, there was no fluctuation observed, and the system was stable till 100 ns (Figure 6(c)).

#### 3.7.4. SASA

To better understand the impact of inhibitors on protein accessible surface area for solvent, we calculated solvent accessible surface area (SASA). The average SASA value of the NDM-1 ligand complex was calculated as 105-119 nm throughout 100 ns. The average SASA values of 118 ± 5 nm for withaferin A, 121 ± 10 nm for mangiferin, and 120 ± 9 nm for D-captopril were observed in 100 ns (Figure 6(d)). All the protein-ligand complexes and apoproteins showed minimum fluctuations during 100 ns. The SASA results showed that active site residues were well exposed to the solvent and readily accessible.

#### 3.7.5. Hydrogen Bond

The number of hydrogen bonds was calculated during simulations for ligand-bound complexes of NDM-1. For NDM-1, an average of 2-3 hydrogen bonds in the NDM-1 ligand-bound complex were observed during 100 ns of simulations. For D-captopril–NDM-1 complex, 3-4 hydrogen bonds were observed in 90-95 ns. Overall, hydrogen bonds between the three ligands had stable interaction with the active site residues of NDM-1 (Figure 6(e)).

### 3.8. MMPBSA Analysis

The binding free energies were calculated using polar and nonpolar energy terms. During the 100 ns MD simulations, the energies associated with the binding of withaferin A, mangiferin, and D-captopril with NDM-1 were calculated. The following energies have been calculated: vdW interaction energy, electrostatic energy, polar solvation energy, SASA energy, and average binding energy (Table 7). NDM-1/ligand complexes and withaferin A/NDM-1 complex showed the lowest binding energy of -96.597 kcal/mol followed by mangiferin/NDM-1 and D-captopril/NDM-1 complexes -168.570 kcal/mol and -132.842 kcal/mol, respectively. The polar and nonpolar energies were distinct in each complex. The lowest polar solvation energy was observed in withaferin A–ligand complexes (39.594 kJ/mol), and the maximum energy was observed in the mangiferin–NDM-1 complex (62.730 kJ/mol). The withaferin A and mangiferin had good binding energies with NDM-1.

### 3.9. Density Functional Theory (DFT) Calculations

#### 3.9.1. HOMO and LUMO Analysis

**Figure 7:**
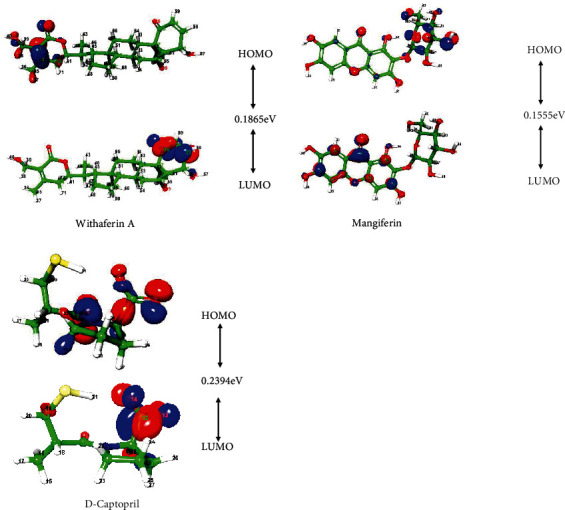
The highest occupied molecular orbital and the lowest unoccupied molecular orbital are plotted on a natural compound.

The properties of the molecular structure were explained using DFT calculations. DFT calculations at the level of B3LYP/6-31G∗∗ were optimized to produce these four molecules. In chemical reactivity, the molecules are sparkly, and the HOMO–LUMO distance increases charge transfer. The molecular orbital interactions with the other species and their energy difference (gap) assist in quantifying the chemical reactivity of the structure that participates in the chemical reactions. Naturally, the electron density is indicated by the intensity of the colour that would reflect the molecule's characteristic feature. The chemical reactivity of HOMO, LUMO, and MESP has been used to analyze molecule parameters. The LUMO is commonly used as an electron acceptor, while the HOMO is used as an electron donor. The stability of the structure was used to analyze the HOMO and LUMO energy gaps. Energy gap levels in the HOMO and LUMO elucidate the fragile essence of reactivity since electrons can be transferred quickly between the energy levels. In HOMO and LUMO, energy gaps of the natural compounds and known inhibitors are 0.1865 eV (withaferin A), 0.1530 eV (mangiferin), and 0.2394 eV (D-captopril), respectively (Table 8). The lesser the energy gap, the more energetically favourable the compound is for chemical reaction. As a result, the values indicate that the molecules have good chemical reactivity. The stability of the compounds was influenced by total energy, dipole moment, HOMO, LUMO, and energy distance. The natural compounds were plotted in the HOMO and LUMO energy gaps (Figure 7). The colour-coding area was red, which indicated the most negative potential region, and blue, which indicated the most positive potential region in the natural compounds.

In Figure 7, the frontier molecular orbital diagrams, the electrons are localized on 4-hydroxy-5,6-epoxy-22-en-1-one moiety in withaferin A and 2-hydroxymethyl-6methloxane-3,4,5-triol in mangiferin in the HOMO structures. The hydroxy groups in withaferin A and mangiferin are readily available to donate electrons to the interaction groups of amino acids at the binding site of NDM-1, based on the HOMO and LUMO structures. The DFT results agree well with the docking results, indicating that withaferin A and mangiferin are the study's best inhibitors. Both withaferin A and mangiferin have the smallest HOMO-LUMO gap among the ligands studied, indicating that the inhibitor's HOMO can transfer electrons to lower-energy LUMO of amino acid residues in the enzyme's active site.

#### 3.9.2. Electrostatic Potential and Dipole Moment

**Figure 8:**
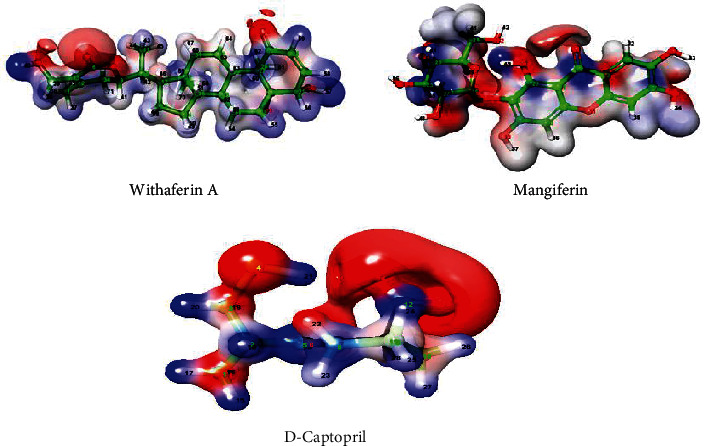
Electrostatic potential map generated for withaferin A and mangiferin, using the density functional theory (DFT) B3LYP/6-31G∗∗ method.

Furthermore, the molecule's electrostatic potential and dipole moment have been proposed to determine the various intermolecular interaction properties and the most suitable regions between ligand and receptor [44]. Based on the electron density distribution on the respective molecular surface, the electrostatic map for withaferin A, mangiferin, and D-captopril is shown in Figure 8. The blue colour region on the electrostatic map indicates the strong electrophilic region of electrons in hydrogen atoms. This was observed for four natural compounds in this analysis. Similarly, the higher nucleophilic regions contributed by electron-rich molecules, such as hydroxy and carbonyl groups in the respective bioactive compounds, were coloured red on electrostatic maps. The measured electrostatic potential map for designated bioactive compounds indicated that highly electronegative atoms, such as oxygen, and highly electropositive atoms, such as carbon atoms bonded to oxygen in cyclic chains and hydrogen atoms, can introduce intermolecular interactions with the active residues of proteins in their vicinity during docking simulations [45]. The highly positive points were located at the hydroxy groups of oxygen atoms, and the highly negative points were located at the hydroxy group of hydrogen for the natural compounds (Figure 8). The natural compounds' high electropositive and electronegative atoms interacted with active site amino acids of NDM-1. The electrostatic potential maximum and minimum ranges for natural compounds and known inhibitors are shown in Table 8, respectively.

### 3.10. Molecular Docking Studies of Other Types of *β*-Lactamase with the Four Natural Compounds

The natural compounds withaferin A and mangiferin were also docked with other types of *β*-lactamases from different organisms to find their broad-spectrum *β*-lactamase activity. The *β*-lactamases, ADC-7 (PDB ID: 6PWL, *A. baumannii*), AmpC (PDB ID: 1KDW, *E. coli*), CTX-M-15 (PDB ID: 4HBT, *E. coli*), KPC-2 (PDB ID: 3RXX, *K. pneumoniae*), OXA-24/40 (PDB ID: 6MPQ, *A. baumannii*), SHV-1 (PDB ID: 3MXR, *K. pneumoniae*), TEM-1 (PDB ID: 1TEM, *E. coli*), and VIM-2 (PDB ID: 2YZ3 *P. putida*), were selected for the Glide docking studies. The previously used protein preparation and Glide protocol in Sections 2.1 and 2.7 were followed for this *β*-lactamase and four natural compounds.

Using XP Glide docking, the natural compounds were docked into their active site region of VIM-2, ADC-7, AmpC, CTX-M-15, KPC-2, SHV-1, and TEM-1 *β*-lactamase. The XP Glide score, Glide energy, and amino acid interactions are shown in Table 9. All four natural compounds had a good docking score and formed hydrogen bonds in the active site amino acids of all seven *β*-lactamases similar to NDM-1. These results indicate that withaferin A and mangiferin have good binding activity with the other types of *β*-lactamases.

## 4. Conclusions

Half of the global occurrence of infections due to *A. baumannii* is attributed to resistance to antibiotics of choice, including CRAB. *A. baumannii* has a solid shield of intrinsic factors and the acquisition of resistance mechanisms, together with working in unison against all known antibiotics of choice for treatment. CRAB is panresistant, often referred to as extensively drug-resistant (XDR). This study focused on identifying potential inhibitors targeting *β*-lactamases with carbapenem as substrate, using molecular docking, molecular dynamic modeling, *in silico* pharmacokinetics, and quantum chemical analysis on a collection of over two hundred phytochemical compounds. The *in silico* screening was done with D-captopril (known inhibitors) against NDM-1 target proteins, respectively, using a comparison of molecular docking scores and hydrogen bond interactions between the active site amino acids of the protein and phytochemicals. The top eight compounds were selected for *in vitro* NDM-1 inhibition assay; withaferin A, mangiferin, mangostin, and rutin showed inhibitory activity against *β*-lactamase and NDM-1 enzymes. Based on the IC_50_ values, withaferin A and mangiferin were subjected to fractional inhibitory concentration assay for ascertaining their mechanistic potential (synergy) with imipenem. The compounds had an FIC index in the range suggestive of possible synergistic mechanism of action potentiating the MIC of imipenem. Further refinement of the results was done using the *in silico* molecular dynamic simulation studies using GROMACS. The RMSD of NDM-1 and compounds and the RMSF and binding energy obtained from MD simulation trajectories strongly indicated that selected molecules have a good potential as *β*-lactamase inhibitors. Quantum chemical analyses revealed that the proposed molecules contain several reaction mechanisms to inhibit lactamase. According to pharmacokinetics analyses, all molecules have a strong absorption, distribution, metabolism, and distribution profile. As a result, it can be concluded that withaferin A and mangiferin could be safe natural potentiators and/or inhibitors substituting the available inhibitors against a wide spectrum *β*-lactamases contributing to carbapenem resistance with a possible use clinically.

## Figures and Tables

**Table 1:** Molecular docking results of natural compounds with NDM-1 protein.

			XP Glide docking			QPLD docking		
S. no	Compounds	Molecular formula	Glide score	Glide energy	Amino acid interaction	Glide score	Glide energy	Amino acid interaction
1	Rutin	C_27_H_30_O_16_	-9.44	-59.59	GLU152, Zn	-9.31	-88.67	HIS250 (PI-PI), LYN211, ASP124, GLU152, ASP223, and Zn
2	Mangiferin	C_19_H_18_O_11_	-9.12	-57.25	ASP223, GLU152, ASP124, and Zn	-10.62	-52.76	GLU152, GLN123, ASN220
3	Phytic acid	C_6_H_18_O_24_P_6_	-7.64	-45.94	Zn, ASP223	-9.24	-59.19	ASN220, ASP124, and Zn
4	Chlorogenic acid methyl ester	C_17_H_20_O_9_	-8.22	-46.44	ASP212, ASN220	-11.01	-58.38	ASP212, LYN211, LYS216, SER217, and ASN220
5	Naringin	C_27_H_32_O_14_	-6.97	-55.64	GLU152, ASP223, and GLN123	-10.87	-68.81	GLN123, TRP93 (PI-PI), LYN211, SER217, and HIS250
6	Quercetrin	C_21_H_20_O_11_	-7.74	-51.90	GLU152	-8.72	-49.49	LYN211
7	Withaferin A	C_28_H_38_O_6_	-5.12	-36.50	ASP124, Zn	-6.21	-43.02	ASP124, Zn
8	Mangostin	C_24_H_26_O_6_	-6.54	-40.17	GLU152, Zn	-7.76	-44.45	ASN220, HIE122 (PI-PI), GLU152, and Zn
9	D-captopril	C_9_H_15_NO_3_S	-5.52	-31.72	Zn, ASN220	-6.28	-42.22	LYS211 (SALT BI), ASP124, ASN220, and Zn
10	Meropenem	C_17_H_25_N_3_O_5_S	-8.77	-41.51	GLU152, ASP223, and Zn	-11.11	-53.66	GLU152, ASP223, and Zn

**Table 2:** SIFt analysis of NDM-1 binding site amino acid interaction with withaferin A.

Amino acid	Side chain	Hydrophobic	Aromatic	Polar	Acceptor	Charged	Backbone
ILE35	1	1	0	0	0	0	0
TRP93	1	1	1	0	0	0	0
HIS122	1	0	0	1	0	0	0
ASP124	1	0	0	1	1	1	0
HIS189	1	0	0	1	0	0	0
SER217	1	0	0	1	0	0	0
GLY219	0	0	0	0	0	0	1
ASN220	1	0	0	1	0	0	1
HIS250	1	0	0	1	0	0	0

**Table 3:** SIFt analysis of NDM-1 binding site amino acids interaction with mangiferin.

Amino acids	Side chain	Hydrophobic	Polar	Acceptor	Charged	Backbone
ILE35	1	1	0	0	0	0
HIS122	1	0	1	0	0	1
GLN123	1	0	1	0	0	1
ASP124	1	0	1	1	1	0
GLU152	1	0	1	1	1	0
HIS189	1	0	1	0	0	0
LYN211	1	0	1	0	0	0
GLY219	0	0	0	0	0	1
ASN220	1	0	1	0	0	0
ASP223	1	0	1	1	1	0
HIS250	1	0	1	0	0	0

**Table 4:** Binding free energy calculation of natural compounds and known inhibitor with NDM-1 protein.

Compounds ID	Binding free energy (kcal/mol) (XP docking)							Binding free energy (kcal/mol) (QPLD)						
	*ΔG* bind	*ΔG* bind Coulomb	*ΔG* bind Covalent	*ΔG* bind Hbond	*ΔG* bind Lipo	*ΔG* bind SolvGB	*ΔG* bind vdW	*ΔG* bind	*ΔG* bind Coulomb	*ΔG* bind covalent	*ΔG* bind Hbond	*ΔG* bind Lipo	*ΔG* bind SolvGB	*ΔG* bind vdW
Withaferin A	-32.20	-42.92	6.82	-0.70	-14.07	78.07	-32.57	-33.49	-39.72	6.82	-0.88	-14.96	75.30	-33.21
Mangiferin	-23.92	-53.47	6.72	-2.91	-12.04	89.30	-40.44	-21.05	-29.32	7.27	-1.26	-12.79	88.86	-47.54
Mangostin	-15.74	-18.50	3.41	-0.91	-15.24	65.68	-42.97	-13.92	-32.57	3.27	-2.28	-15.42	63.13	-46.23
Rutin	-24.32	-50.66	3.41	-2.72	-10.54	104.10	-44.10	-27.18	-51.41	2.27	-5.48	-15.97	46.60	-48.59
D-captopril	-11.93	-67.53	5.68	-0.45	-4.93	83.25	-22.25	-18.57	-12.76	6.82	-0.35	-5.002	102.46	-22.91

**Table 5:** Fractional inhibitory concentration (FIC) and FIC indices (FICIs) of combination of withaferin A and mangiferin with imipenem against *A. baumannii* MDR strain.

*A. baumannii* MDR strain	FIC	FIC index	Interpretation
Imipenem with withaferin	0.0625	0.3125	Synergy
Withaferin with imipenem	0.25		
Imipenem with mangiferin	0.125	0.625	Synergy
Mangiferin with imipenem	0.5		

**Table 6:** ADME properties of four natural compounds.

Compounds	#rotor	mol MW	donorHB	accptHB	QPpolrz	QPlogPC16	QPlogPoct	QPlogPw	QPlogPo/w	QPlogS	QPlogKp	QPlogKhsa	PSA
Accepted range	(1-15)	(130.0–725.0)	(0.0–6.0)	(2.0–20.0)	(13.0–70.0)	(4.0–18.0)	(8.0–35.0)	(4.0–45.0)	(−2.0–6.5)	(−6.5–0.5)	(−8.0–−1.0)	(−1.5–1.5)	(7.0–200.0)
Mangiferin	11	438.344	6	13.75	34.718	14.346	30.63	26.29	-1.82	-2.282	-6.727	-1.013	190.756
Withaferin A	5	470.605	1	9.4	47.967	13.214	23.019	12.717	3.117	-4.997	-3.993	0.385	114.741
Mangostin	8	380.44	2	3.75	39.477	12.498	18.024	8.63	4.449	-5.672	-2.54	0.779	92.69
Rutin	15	610.524	6	18	48.459	17.033	41.62	35.966	-2.558	-2.242	-7.331	-1.298	273.511

**Table 7:** Binding free energy and interaction energy of NDM-1–ligand complexes calculated using the MM-PBSA approach.

Target	ID	Binding energy (kJ/mol)	Van der Waal energy (kJ/mol)	Electrostatic energy (kJ/mol)	Polar solvation energy (kJ/mol)	SASA energy (kJ/mol)
NDM-1	Withaferin A	−96.597 ± 56.626	−112.747 ± 67.710	−12.105 ± 12.260	39.594 ± 38.035	−11.339 ± 6.804
	Mangiferin	−168.570 ± 48.904	−97.906 ± 44.098	−114.624 ± 77.901	62.730 ± 31.574	−10.878 ± 5.269
	D-captopril	−132.842 ± 35.026	−60.775 ± 19.936	−99.864 ± 55.580	101.701 ± 66.646	−7.948 ± 2.811

**Table 8:** HOMO, LUMO, HELG, ESP, and solvation energy parameter of the withaferin A, mangiferin, and D-captopril.

S. no	Compound IDs	HOMO (eV)	LUMO (eV)	HLG (eV)	QM dipole (debye)	Solvation energy (kcal/mol)	ESP min kcal/mol	ESP max kcal/mol
1	Withaferin A	-0.25442	-0.06792	0.1865	8.9441	-23.82	-62.87	63.53
2	Mangiferin	-0.21798	-0.06246	0.1555	7.6612	-15.80	-74.91	79.73
3	D-captopril	-0.2394	-0.0058	0.2394	11.2215	-71.45	-79.28	76.73

**Table 9:** The binding interactions of natural compounds in the active site residues of other *β*-lactamase enzymes.

Compounds	Beta-lactamase	Glide score	Glide energy	Glide EvdW	Glide Ecoul	Glide emodel	Amino acid interactions
Withaferin A	AMPC	-4.606	-38.022	-23.74	-14.282	-50.842	ARG204, GLN120, ASN289, and ASN346
	TEM-1	-2.791	-31.98	-17.237	-14.743	-34.431	GLU110, SER106, and VAL216
	VIM-2	-3.848	-35.208	-25.42	-9.788	-39.222	ASP97, ASN190
	SHV-1	-4.145	-31.208	-23.642	-7.566	-36.326	MET129
	KPC-2	-3.665	-32.831	-28.41	-4.422	-42.998	CYS238
	OXA 24/40	-5.68	-38.15	-35.609	-2.543	-37.991	SER128, SER81
	CTX-M-15	-4.1	-34.691	-25.794	-8.896	-41.456	GLY238
	ADC-7	-3.562	-30.258	-23.526	-6.732	-35.533	ASN204, GLN120, and PHE121
Mangiferin	AMPC	-7.848	-49.667	-30.064	-19.603	-62.404	ASN343, VAL121, and ASP123
	TEM-1	-4.079	-29.904	-20.442	-9.462	-40.812	ALA237, GLU104
	VIM-2	-6.117	-46.288	-20.793	-25.494	-56.383	TRP67, GLU126, and ASP43
	SHV-1	-7.653	-43.892	-28.665	-15.228	-57.531	TYR105, SER130, ASN276, and VAL216
	OXA 24/40	-10.05	-50.28	-34.581	-15.707	-69.695	ARG261, LEU127, and ALA126
	KPC-2	-6.293	-36.214	-25.905	-10.309	-47.456	TYR129, CYS238
	CTX-M-15	-6.117	-55.386	-26.468	-28.918	-84.69	ASN132
	ADC-7	-4.738	-33.168	-15.139	-18.029	-40.601	GLU289, ASN287, ASN343, and GLU344

## Data Availability

Data are contained within the article.

## Supplementary Materials

Supplementary 1 Supplementary Figure 1: Ramachandran plots of the NDM-1 3D models. Ramachandran plots of the NDM-1 3D models built by homology modeling obtained by PROCHECK. 89.7%, residues in most favoured regions; 9.8%, residues in additional allowed regions; 0.0%, residues in generously allowed regions; and 0.5%, residues in disallowed regions.

Supplementary 2 Supplementary Figure 2: docking interaction fingerprints of natural compounds with active site residues of NDM-1 protein.

Supplementary 3 Supplementary Figure 3: validation of docking protocol by extracting and redocking the crystal structure bound inhibitor (D-captopril) at the active site of NDM-1 using XP docking. The redocked of D-captopril shows that it was docked into a similar position and amino acid interaction at the active site of NDM-1 in the docked conformations, as was present in the X-ray crystal structure.
