# Supplementary material for: In Silico and In Vitro Screening of Natural Compounds as Broad-Spectrum β-Lactamase Inhibitors against Acinetobacter baumannii New Delhi Metallo-β-lactamase-1 (NDM-1)
Source: Biomed Res Int. 2022 Mar 10;2022:4230788. doi: 10.1155/2022/4230788 (PMC8966755; doi:10.1155/2022/4230788)
Supplement: Supplementary 1 — Supplementary Figure 1: Ramachandran plots of the NDM-1 3D models. Ramachandran plots of the NDM-1 3D models built by homology modeling obtained by PROCHECK. 89.7%, residues in most favoured regions; 9.8%, residues in additional allowed regions; 0.0%, residues in generously allowed regions; and 0.5%, residues in disallowed regions. [file 4230788.f1.docx]

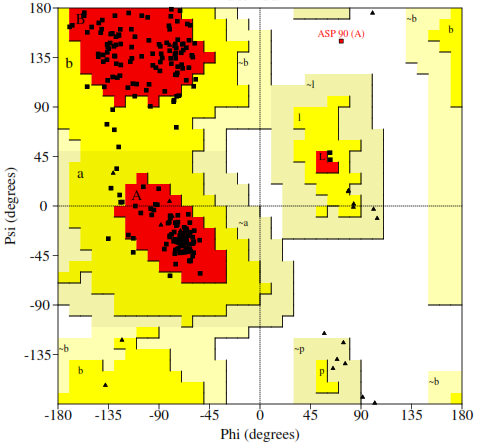


**Supplementary figure 1**. Ramachandran plots of the NDM-1 3D models. Ramachandran plots of the NDM-1 3D models built by homology modeling obtained by PROCHECK. 89.7%, residues in most favoured regions; 9.8%, residues in additional allowed regions; 0.0%, residues in generously allowed regions and 0.5%, residues in disallowed regions.
