# Supplementary material for: In Silico and In Vitro Screening of Natural Compounds as Broad-Spectrum β-Lactamase Inhibitors against Acinetobacter baumannii New Delhi Metallo-β-lactamase-1 (NDM-1)
Source: Biomed Res Int. 2022 Mar 10;2022:4230788. doi: 10.1155/2022/4230788 (PMC8966755; doi:10.1155/2022/4230788)

**Supplementary figure 2.** Docking interactions fingerprints of natural compounds with active site residues of NDM-1 protein.

| Compounds | XP Glide Docking | QPLD Docking |
| --- | --- | --- |
| Rutin | 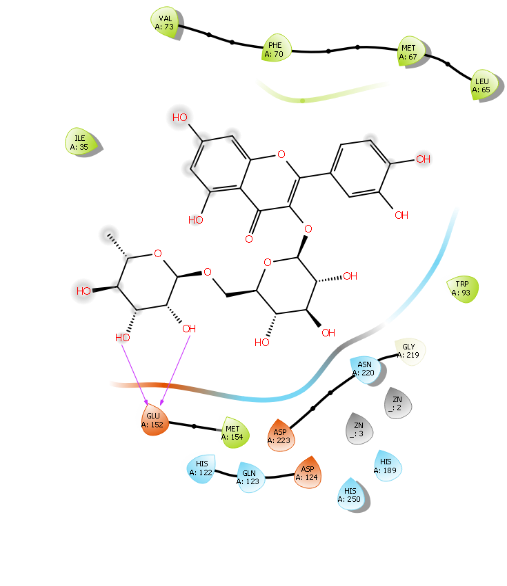 | 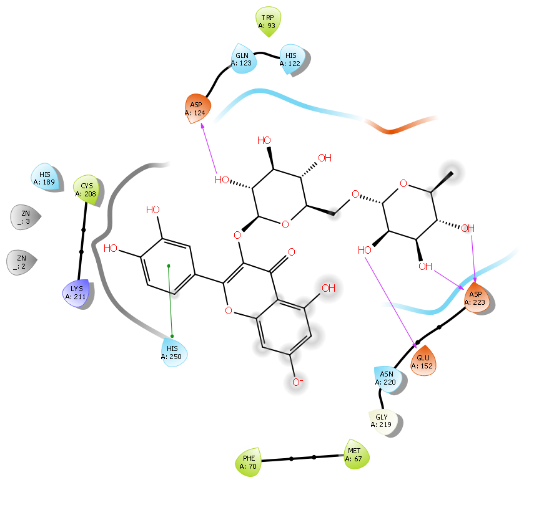 |
| Mangiferin | 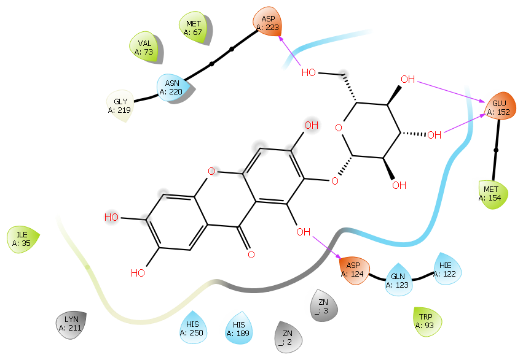 | 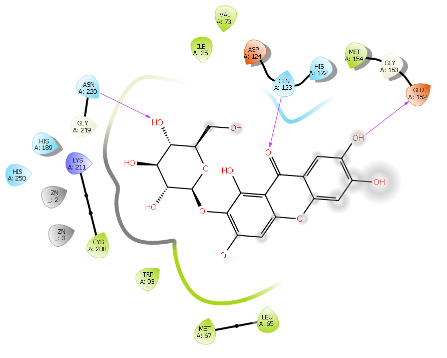 |
| Withaferin A | 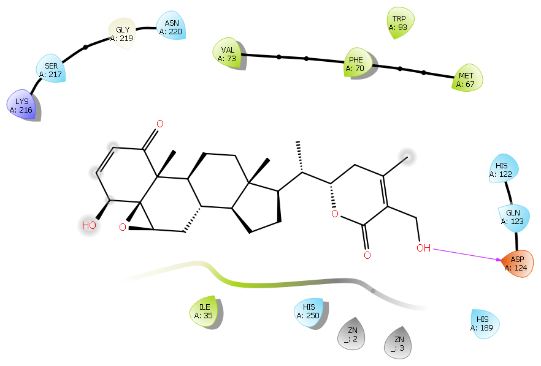 | 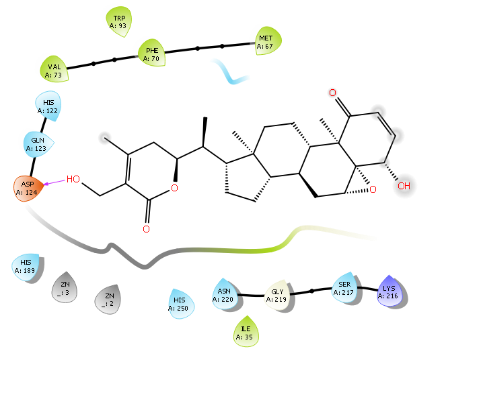 |
| Mangostin | 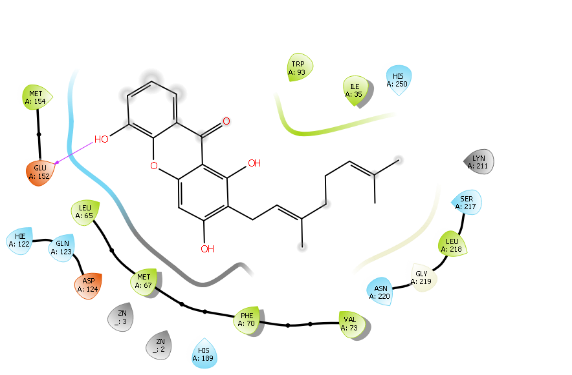 | 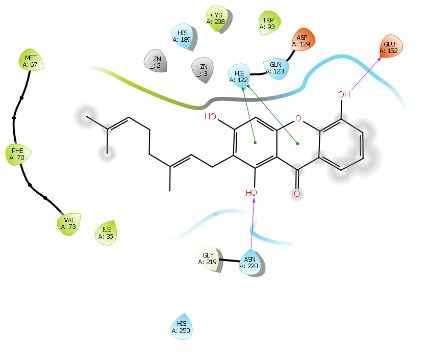 |
| D-Captopril | 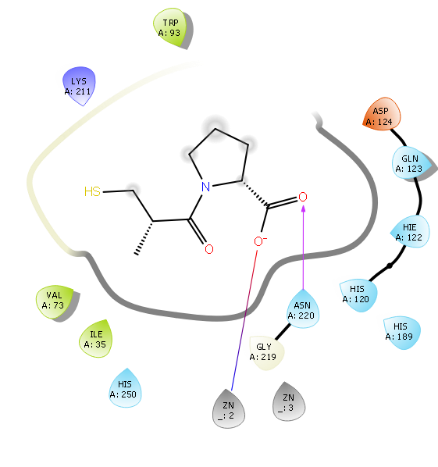 | 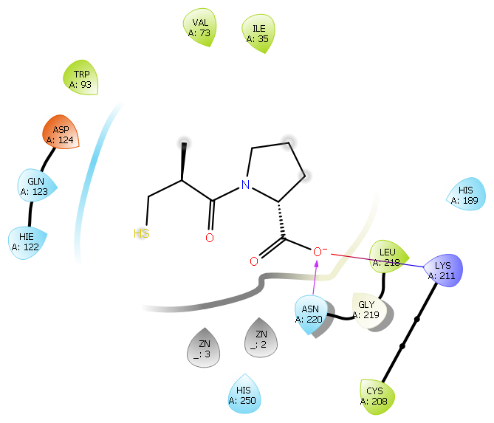 |


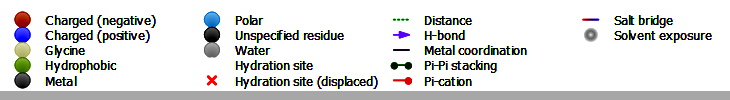

Supplement: Supplementary 2 — Supplementary Figure 2: docking interaction fingerprints of natural compounds with active site residues of NDM-1 protein. [file 4230788.f2.docx]
