# Supplementary material for: In Silico and In Vitro Screening of Natural Compounds as Broad-Spectrum β-Lactamase Inhibitors against Acinetobacter baumannii New Delhi Metallo-β-lactamase-1 (NDM-1)
Source: Biomed Res Int. 2022 Mar 10;2022:4230788. doi: 10.1155/2022/4230788 (PMC8966755; doi:10.1155/2022/4230788)

**Supplementary Figure 3:** Validation of docking protocol by extracting and re-docking the crystal structure bound inhibitor (D-Captopril) at the active site of NDM-1 using XP docking. The redocked of D-Captopril shows that it was docked into a similar position and amino acid interaction at the active site of NDM-1 in the docked conformations, as was present in the X-ray crystal structure.

| 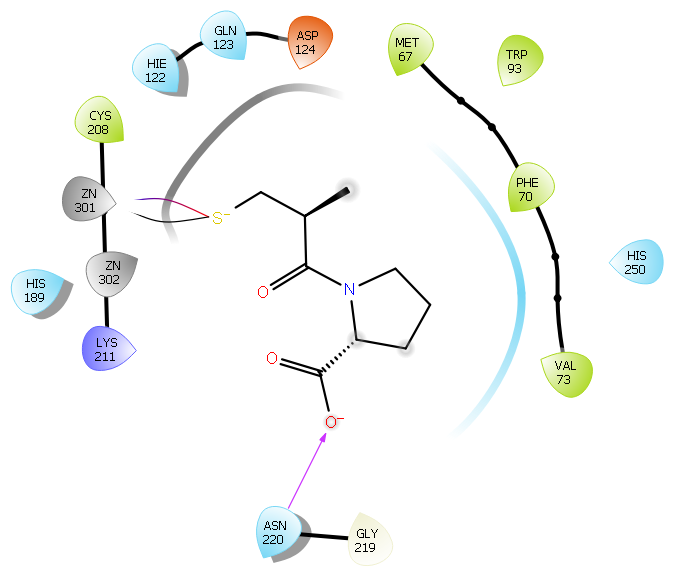 | 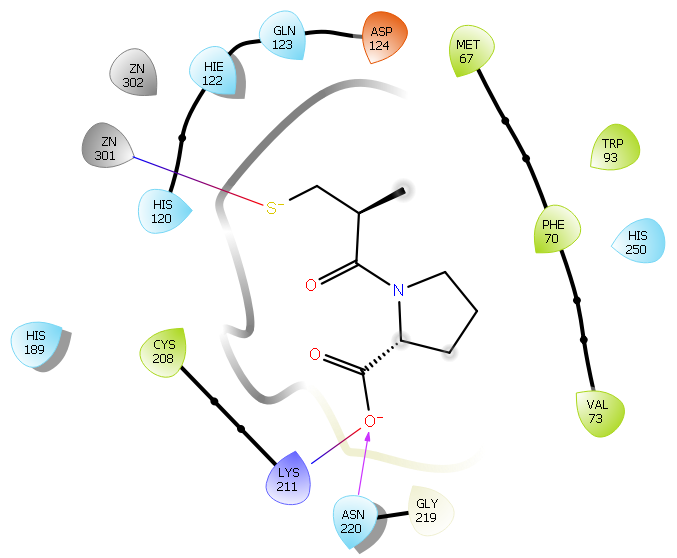 |
| --- | --- |
| Crystal structure - D-Captopril | Redocked - D-Captopril |


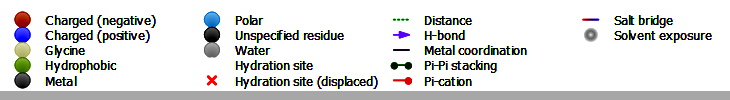

Supplement: Supplementary 3 — Supplementary Figure 3: validation of docking protocol by extracting and redocking the crystal structure bound inhibitor (D-captopril) at the active site of NDM-1 using XP docking. The redocked of D-captopril shows that it was docked into a similar position and amino acid interaction at the active site of NDM-1 in the docked conformations, as was present in the X-ray crystal structure. [file 4230788.f3.docx]
